# Essential Amino Acid Supplementation May Attenuate Systemic Inflammation and Improve Hypoalbuminemia in Subacute Hemiplegic Stroke Patients

**DOI:** 10.3390/metabo15090626

**Published:** 2025-09-19

**Authors:** Mirella Boselli, Roberto Aquilani, Roberto Maestri, Paolo Iadarola, Alessandro Magistroni, Chiara Ferretti, Antonia Pierobon, Matteo Cotta Ramusino, Alfredo Costa, Daniela Buonocore, Marco Peviani, Federica Boschi, Manuela Verri

**Affiliations:** 1 Neuromotor Rehabilitation Unit, Istituti Clinici Scientifici Maugeri IRCCS, Montescano Institute, 27040 Montescano, Italy; mirella.boselli@icsmaugeri.it (M.B.); alessandro.magistroni@icsmaugeri.it (A.M.); chiara.ferretti@icsmaugeri.it (C.F.); 2 Department of Biology and Biotechnology “Lazzaro Spallanzani”, University of Pavia, 27100 Pavia, Italy; dottore.aquilani@gmail.com (R.A.); paolo.iadarola@unipv.it (P.I.); daniela.buonocore@unipv.it (D.B.); marco.peviani@unipv.it (M.P.); 3 Department of Biomedical Engineering, Istituti Clinici Scientifici Maugeri IRCCS, Montescano Institute, 27040 Montescano, Italy; roberto.maestri@icsmaugeri.it; 4 Psychology Unit, Istituti Clinici Scientifici Maugeri IRCCS, Montescano Institute, 27040 Montescano, Italy; antonia.pierobon@icsmaugeri.it; 5 Unit of Clinical Neuroscience of Dementias, Dementia Research Center (DRC), IRCCS Mondino Foundation, 27100 Pavia, Italy; matteo.cottaramusino@mondino.it; 6 Department of Brain and Behavioural Sciences, University of Pavia, 27100 Pavia, Italy; alfredo.costa@mondino.it; 7 Unit of Behavioural Neurology, IRCCS Mondino Foundation, 27100 Pavia, Italy; 8 Department of Drug Sciences, University of Pavia, 27100 Pavia, Italy; federica.boschi@unipv.it

**Keywords:** subacute stroke, systemic inflammation, hypoalbuminemia, essential amino acid supplementation

## Abstract

**Background:** Post-stroke inflammation and hypoalbuminemia can negatively affect neurocognitive recovery. This study evaluated whether oral amino acid (AA) supplementation with prevalently essential amino acids (EAAs, 82.1%) could improve inflammation and albumin levels in post-stroke patients undergoing neurorehabilitation. **Methods:** Sixty-four patients with subacute stroke (less than three 3 months from acute event) and elevated inflammation markers (C-reactive protein, CRP > 0.5 mg/dL) were enrolled. All underwent anthropometric assessments and blood tests for CRP (normal value < 0.5 mg/dL), albumin (normal range: 3.5–4.76 g/dL), prealbumin (18–32 mg/dL), and white blood cell count. Participants were randomly assigned to receive either oral EAAs (8.4 g/day) or placebo (maltodextrin, 8.4 g/day) for 55 days. Measurements were taken at baseline (T0) and at discharge (T1), approximately two months later. **Results:** At baseline, both groups had comparable levels of systemic inflammation, albumin and prealbumin: CRP, 2.13 ± 1.82 mg/dL (placebo) vs. 2.89 ± 2.12 mg/dL (EAAs), *p* = 0.13; albumin, 3.10 ± 0.46 g/dL (placebo) vs. 3.07 ± 0.57 g/dL (EAAs), *p* = 0.82; prealbumin, 18.3 ± 6.2 mg/dL (placebo) vs. 16.9 ± 3.9 mg/dL (EAAs), *p* = 0.28. During rehabilitation, only the EAA group showed significant reductions in CRP (*p* = 0.036 vs. placebo) and improvements in albumin (*p* = 0.033 vs. placebo) and prealbumin levels (*p* = 0.05 vs. placebo). However, full normalization of CRP and albumin was not achieved. **Conclusions:** This study demonstrates that a physiological dose of supplemented EAAs may attenuate, but not fully resolve, post-stroke inflammation and hypoalbuminemia. Further research is needed to determine whether higher EAA doses and/or modifications in EAA composition could enhance or normalize systemic inflammation.

## 1. Introduction

Stroke is the leading cause of long-term disability among survivors of this acute event. One year after a stroke, approximately one-third of patients remain permanently disabled [1,2], and about two-thirds do not achieve full recovery [3]. Additionally, one-third are unable to walk without assistance and exhibit reduced gait efficiency due to the high energy cost of walking [4,5]. Numerous trials aimed at improving upper and lower limb motor function through rehabilitation in post-stroke patients have not demonstrated the superiority of training, technological aids, pharmacological treatments, or neuromodulation techniques over standard care [6]. Some observational studies and clinical trials have reported neurocognitive benefits in post-stroke patients supplemented with proteins or amino acids [7], as well as vitamins and minerals [8]. Protein (20 g protein) and energy (250 kcal) supplementation in rehabilitative stroke patients enhanced the spontaneous recovery of neurological alterations as assessed by the National Institute of Health Stroke Scale [7]. Zinc supplementation (10 mg/day) in ischemic stroke patients to normalize zinc intake enhanced neurological retrieval [9]. Regarding oral essential amino acid (EAA) supplementation, one study demonstrated that supplementation with leucine-rich amino acids improved patients’ physical capacity as measured by the Functional Independence Measure (FIM) [10]. Another study demonstrated that administering the EAA branched-chain amino acids (BCAAs: leucine, valine, isoleucine) at breakfast in combination with a structured exercise regimen enhanced functional recovery in stroke patients [11,12]. An earlier study reported that oral EAA supplementation (8 g/day) to dysphagic subacute stroke patients shifted the metabolic state of the unaffected arm muscle from hypercatabolism to anabolism [13], thereby preventing muscle breakdown and potentially enhancing functional independence post-stroke [14]. However, the current evidence is insufficient to recommend nutritional or metabolic interventions for the entire post-stroke rehabilitation population as a means to improve functional outcomes [8,15].

At present, beyond standard rehabilitative care, no established treatment has been shown to consistently improve functional outcomes in post-stroke patients. An emerging area of interest is the role of post-stroke inflammation (PSI), which may contribute significantly to disability in stroke survivors. PSI has been identified as a major factor in the development of fatigue, depression, cognitive decline, and neuropsychiatric disturbances [16], all of which pose serious barriers to rehabilitation and recovery [17,18]. Fatigue, in particular, hampers physical activity [19], limits participation in rehabilitation [20], delays neurological recovery [21], impairs socialization [22], and diminishes quality of life [21,23], while also being associated with increased mortality [21]. The use of cytokine antagonists has been shown to improve stroke-related fatigue [24,25,26,27,28,29], highlighting inflammation as a key contributor to this condition. Depression affects approximately one-third of stroke survivors and is increasingly recognized as being driven, in part, by inflammatory mechanisms [18,30]. Post-stroke depression interferes with both cognitive and functional recovery, reduces engagement in rehabilitation, and impairs neuroplasticity [31,32], thereby contributing to poorer overall outcomes [18]. Post-stroke inflammation significantly impacts cognitive function in stroke survivors [33], increasing the risk of developing dementia [34] and contributing to brain atrophy [35,36,37,38,39]. Two key biological mechanisms make PSI a particularly serious complication: the establishment of a vicious cycle between central and peripheral inflammation [16,40] and the prolonged duration of the inflammatory response. Cerebral artery occlusion initiates brain inflammation, which subsequently triggers a systemic inflammatory response within hours of the stroke event [16,41,42,43,44]. This inflammatory state can persist for months after the acute event, both in the brain and systemically [45,46]. Elevated levels of pro-inflammatory cytokines—particularly IL-1β and IL-6—have been detected up to three months post-stroke [7,47,48], with higher concentrations observed in patients who experienced larger strokes [47]. Serum C-reactive protein (CRP), a primary clinical marker of inflammation, remains elevated for up to three months following an acute ischemic stroke [49,50,51,52]. CRP levels tend to be higher in patients with large-vessel occlusion compared to those with small-vessel stroke [50]. Circulating levels of IL-4 and IFN-γ also remain elevated up to three months after the acute event, regardless of stroke etiology [53]. This prolonged inflammatory state may be further sustained by changes in gut microbiota triggered by acute stroke itself [54,55] and by post-stroke infections [56,57]. Collectively, these studies suggest that ischemic stroke patients experience a persistent disruption of peripheral immune responses, characterized by a dominance of innate (pro-inflammatory) over adaptive (repair-oriented) immunity. Despite this understanding, current immune therapies aimed at reducing inflammation and improving outcomes have not proven effective in clinical trials [58], and no immunomodulatory drugs for stroke have been approved to date. However, treatment with a sphingosine-1-phosphate receptor modulator showed promise in a small patient cohort, improving functional outcomes at 90 days post-stroke [58,59]. In experimental models of ischemic stroke in rats, DL-3-n-butylphthalide (NBP) and edaravone dexborneol (Eda-Dex) have been shown to improve neurological function and alleviate cognitive impairment by synergistically inhibiting inflammation and oxidative stress [60].

In the present study, we hypothesized that supplementation with a mixture of free amino acids (AAs), consisting of 82.1% EAAs, may help reduce post-stroke inflammation (PSI) in patients undergoing rehabilitation. EAAs are natural substrates known to stimulate protein synthesis, support mitochondrial function, and exert anti-inflammatory effects. These properties have been documented in several clinical contexts: during acute infections in otherwise healthy individuals [61]; in sarcopenic elderly patients with diabetes undergoing hypoglycemic therapy, where EAA supplementation reduced plasma TNF-α levels [62]; and in dysphagic stroke patients undergoing rehabilitation, where EAAs decreased CRP levels and improved swallowing function [63]. Additionally, in patients with chronic kidney disease, EAAs have been shown to reduce gut inflammation, as indicated by lower fecal calprotectin levels [64]. Reducing systemic inflammation may also improve clinical outcomes by increasing levels of circulating albumin (Alb)—a negative acute-phase reactant known to decrease during inflammation [52,65,66]. In subacute stroke patients, improved inflammatory status has been positively correlated with higher Alb concentrations [66]. Alb, in turn, may enhance neurocognitive recovery, as suggested by preclinical studies showing its neuroprotective effects in models of transient focal ischemia [67,68,69], global ischemia [70], and traumatic brain injury [71].

In this prospective, interventional, double-blind, randomized controlled trial, patients with subacute stroke (<3 months from the acute event) and evidence of systemic inflammation were recruited and administered 8.4 g/day of EAAs in pharmaceutical formulation.

The primary objective was to determine whether EAA supplementation leads to a significant reduction in baseline CRP levels. The secondary objective was to evaluate improvements in serum albumin levels among patients presenting with hypoalbuminemia.

The potential impact of normalized inflammatory markers and albumin levels on neurocognitive recovery is reserved for future investigation. The aim of this initial study was to evaluate the extent to which inflammation and hypoalbuminemia may be improved through the administration of natural metabolic substrates that can be safely and easily modulated exogenously.

## 2. Materials and Methods

### 2.1. Population

**Figure 1:**
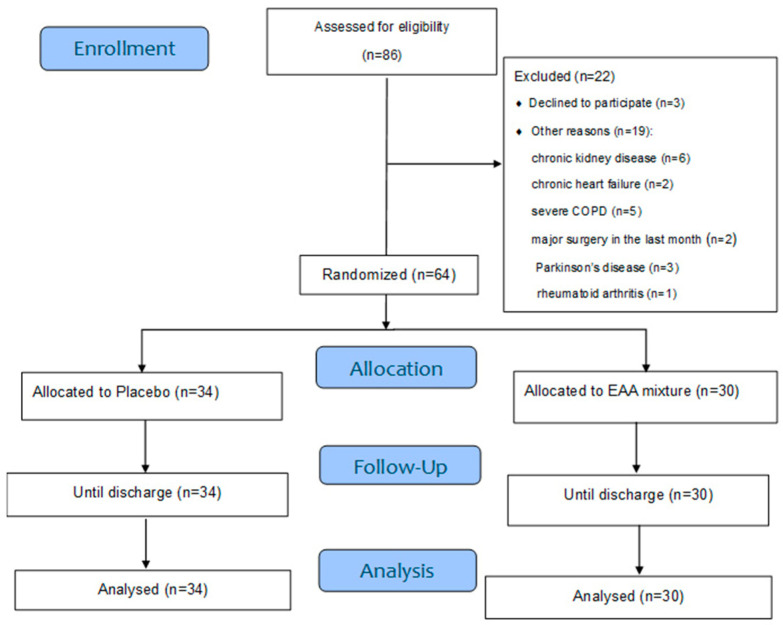
Design and flow of the study. Abbreviations:COPD, Chronic Obstructive Pulmonary Disease; EAA, Essential Amino Acid.

Eighty-six subacute hemiplegic stroke patients (<3 months from the acute cerebrovascular event) [7] with elevated circulating C-reactive protein (CRP) levels (>0.5 mg/dL), as reported in hospital discharge summaries, were screened for eligibility at the Neurorehabilitation Centre of Montescano (Pavia, Italy). Of these, 64 patients met inclusion criteria and were ultimately analyzed (Figure 1). Patient recruitment occurred between 31 December 2019, and 31 December 2024, across two distinct periods: pre-pandemic and post-COVID-19 pandemic. Recruitment was suspended for three years during the pandemic, as the rehabilitation unit was repurposed for the care of COVID-19 and post-COVID patients. Given the persistence of post-stroke inflammation for up to three months following the index event [7,47,48], and in line with the primary objective of the present study, patients were eligible for recruitment up to three months post-stroke, regardless of the exact timing of the event. The patients included in the study had experienced an acute cerebrovascular event a mean of 18 ± 3.5 days prior to admission to the rehabilitation department. All patients with signs of inflammation were bedridden and admitted from neurological departments or stroke units. These clinical settings are specifically dedicated to the diagnosis and treatment of acute stroke and also provide initial rehabilitation interventions prior to patients’ transfer to rehabilitation institutes or wards.

Diagnosis of stroke was confirmed by computed tomography, which revealed ischemic lesions in 82.8% of patients and haemorrhagic lesions in 17.2%. Inclusion criteria were male and female patients over 40 years of age who had experienced an ischemic or hemorrhagic stroke within three months prior to recruitment and exhibited systemic inflammation, indicated by elevated serum CRP levels. Exclusion criteria included chronic kidney disease, chronic heart failure, severe chronic obstructive pulmonary disease (COPD), recent major surgery (within one month), Parkinson’s disease, and collagenopathies (further detailed in the flow diagram, Figure 1).

At admission, nutritional support was provided either via nasogastric tube (n = 13; 20.3%) or through standard oral/modified diets (n = 51; 79.7%).

### 2.2. Procedures

1. Variables of routine assessment

At enrolment, patients underwent the following assessments:

(a) Anthropometric Measurements. Body weight (BW, kg) and height (m) were recorded, with height estimated from knee height [72]. Body Mass Index (BMI) was then calculated as weight (kg) divided by height squared (m^2^).

(b) Blood Tests. On the morning following admission (8:00 a.m.), after an overnight fast, venous blood samples were collected to assess routine biochemical and inflammatory markers. The following parameters were measured:

• C-Reactive Protein (CRP): A primary marker of inflammation (normal value < 0.5 mg/dL).
• Serum proteins:
  ○ Negative acute-phase proteins:
    ▪ Albumin (normal value: 3.5–4.76 g/dL)
    ▪ Prealbumin (normal value: 18–32 mg/dL)
    ▪ Transferrin (normal value: 202–364 mg/dL)
  ○ Positive acute-phase protein:
    ▪ Fibrinogen (normal value: 230–550 mg/dL).
• White Blood Cell (WBC) profile: Total white blood cell count (TWBC), as well as neutrophil and monocyte counts, were evaluated as non-specific indicators of inflammation. The neutrophil-to-lymphocyte ratio (N/L ratio), with a laboratory reference range of 1.5–3.0, was used as an indicator of the balance between innate and adaptive immune responses [73].
• Albumin/CRP ratio: This ratio was calculated to adjust for the inflammatory component influencing serum albumin levels and to provide a more specific index of nutritional and inflammatory status.

All anthropometric and bio humoral variables were assessed at admission (baseline; Time 0, T0) and again at discharge (Time 1, T1), approximately two months after admission.

2. Functional status

Patients’ functional abilities were assessed using the Functional Independence Measure (FIM), as previously described [56]. The FIM is an 18-item scale designed to evaluate a patient’s level of physical and cognitive independence. It includes two distinct subscales:

• Motor FIM (M-FIM): Assesses activities such as feeding, grooming, dressing, toileting, and mobility. Score range: 13 to 91.
• Cognitive FIM (C-FIM): Assesses cognitive functions including communication and social cognition. Score range: 5 to 35.

For simplicity, in this study we refer to Total FIM (T-FIM), the sum of M-FIM and C-FIM scores. Total range: 18 (complete dependence) to 126 (complete independence).

FIM assessments were conducted at baseline and repeated biweekly until discharge to monitor progress in functional recovery.

3. Patient randomization

Following baseline assessments, patients were randomized to receive either an EAA supplement or a placebo (maltodextrin) for a duration of 55 days. The composition of the EAA mixture is detailed in Table 1, while Table 2 presents the demographic, anthropometric characteristics and comorbidities of the randomized groups.

Randomization was performed using a computer-generated list created with SAS statistical software version 9.4 (SAS Institute Inc., Cary, NC, USA). Treatment arms were labelled as “A” and “B” to maintain blinding. The randomization list was made available only to the physician (Mirella Boselli) and the hospital pharmacists. Patients were sequentially assigned to treatment A or B according to the list.

The investigator responsible for interpreting the study results (Roberto Aquilani) remained blinded to treatment allocation throughout the study.

The EAA group received 8.4 g/day of free-form EAAs (Amino-Ther Pro, Professional Dietetics, Milan, Italy) administered as 4.2 g in the morning and 4.2 g in the afternoon, each dissolved in 150 mL of water, until patient discharge.

The placebo group (Plac) received an isocaloric maltodextrin supplement (8.4 g/day) matched in appearance, taste, and volume to the EAA mixture. In both groups, rehabilitation nurses supervised and assisted patients during the intake of the supplement or placebo to ensure adherence and monitor for potential adverse effects.

4. Rehabilitation protocol

All patients participated in a standardized multidisciplinary rehabilitation programme throughout their hospital stay. Neuromotor therapy was administered for 60 min per day, five days per week, with an additional 30 min session on Saturdays.

In addition, patients received approximately 3 h per day of combined occupational therapy and speech/neuropsychological therapy, five days per week [13].

The rehabilitation regimen included a combination of:

• Passive, active, and active-assistive range of motion exercises;
• Coordination and facilitation techniques targeting the paretic (contralateral) limbs;
• Trunk stabilization and strengthening exercises;
• Active exercises for the unaffected limbs;
• Ambulation training using assistive devices or therapist support as needed.

This comprehensive approach aimed to promote motor recovery, functional independence, and cognitive-communicative improvements.

All participants, or their legal caregivers (when applicable) provided written informed consent prior to enrolment, after receiving a full explanation of the study objectives and procedures. The study was conducted in accordance with the ethical principles outlined in the Declaration of Helsinki and was approved by the ICS Maugeri Ethics Committee (approval date: 16 May 2017; protocol No. 2112 CE).

The study protocol was finalized and published prior to patient enrolment. Although formal trial registration was waived, the study protocol was documented and approved in accordance with institutional and national regulatory requirements.

### 2.3. Objectives of the Study

The primary objective of the study was to determine whether EAA supplementation leads to a significant reduction in baseline CRP levels.

The secondary objective was to assess improvements in serum albumin (Alb) levels among patients with hypoalbuminemia.

### 2.4. Statistical Analysis

#### 2.4.1. Sample Size Estimation

We computed the minimum sample size required to detect a difference of at least 1.6 mg/dL in the change (discharge—baseline) of serum CRP between EAA-supplemented and placebo patients (corresponding to a large effect size, Cohen’s *d* = 0.75), with 80% power and a two-tailed type I error rate of 0.05. The resulting sample size was 58 patients, with 29 in the EAA-supplemented group and 29 in the placebo group. Computations were performed using the ‘proc power’ of the SAS statistical package (SAS/STAT, release 9.4, SAS Institute Inc., Cary, NC, USA).

#### 2.4.2. Between-Group Comparisons

The distribution of continuous variables was assessed for normality using the Shapiro–Wilk test. Although some variables did not meet the assumption of normality, the deviations were minor. Consequently, continuous variables were summarized using mean ± standard deviation (SD). Hypothesis testing primarily employed parametric methods, with non-parametric tests used for confirmation. Categorical data were described using absolute and relative frequencies (%).

Between-group comparisons for continuous variables were conducted using independent samples *t*-tests, with confirmation via the Mann–Whitney U test. Within-group comparisons were performed using paired *t*-tests, verified with the Wilcoxon signed-rank test. Comparisons of categorical variables were made using the chi-square test or Fisher’s exact test, as appropriate.

To examine whether there were differences in the temporal evolution (T1 vs. T0) of the variables between the EAA and placebo groups, we calculated the change (delta) between T1 and T0 for each variable and compared these differences between the groups.

Associations between pairs of variables were evaluated using Spearman’s rank correlation coefficient (Spearman’s r).

All statistical tests were two-tailed, and a *p*-value of less than 0.05 was considered statistically significant. All analyses were conducted using the SAS/STAT software package, version 9.4 (SAS Institute Inc., Cary, NC, USA).

## 3. Results

At baseline (T0) (Table 3), all inflamed patients enrolled in the study presented with elevated serum CRP levels, a predominance of innate over adaptive immunity (as indicated by a high N/L ratio), hypoalbuminemia and low prealbumin levels. From a neurofunctional perspective, patients exhibited significant impairments in M-FIM, C-FIM, and overall T-FIM.

Following randomization, the patient subgroups showed comparable clinical characteristics (Table 2), including (Table 4) similar serum CRP levels, hypoalbuminemia and low prealbumin levels. The subgroups also demonstrated similar levels of M-FIM, C-FIM, and T-FIM.

During the rehabilitation phase (Table 5) both the placebo and EAA groups showed increases in circulating Alb levels, however the improvement was statistically significant only in the EAA group (*p* = 0.033). Furthermore, a significant reduction in systemic inflammation, indicated by decreased serum CRP levels (*p* = 0.036) was observed exclusively in the EAA-treated patients. EAA supplementation also led to a statistically significant improvement in prealbumin levels (*p* = 0.05). Changes in body weight, haemoglobin, creatinine, blood urea, TWBC, and white cell subpopulations were comparable between the placebo and EAA groups. Regarding neurofunctional outcomes, both patient subgroups demonstrated similar rates of recovery in motor and cognitive functions, as well as in overall functional disability.

**Figure 2:**
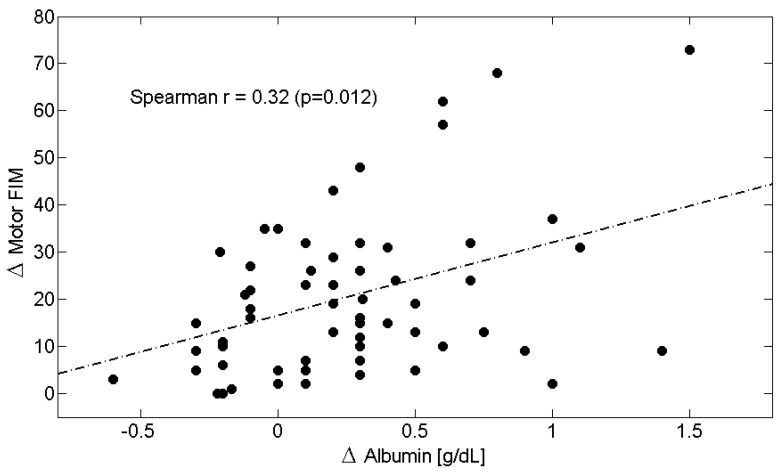
Scatterplot showing the relationship between the change (Δ = T1–T0) in Motor FIM scores and the change in Albumin in all patients. The linear regression line is shown as a dash-dotted line. Spearman’s correlation coefficient and *p*-value are also reported. Abbreviations: FIM, Functional Independence Measure.

**Figure 3:**
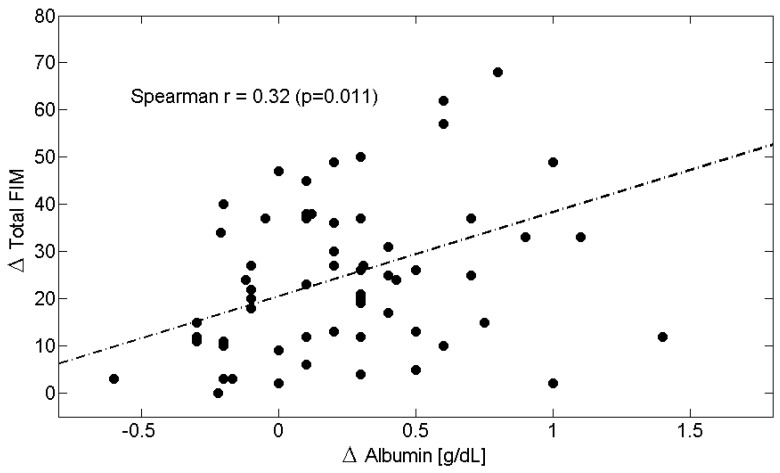
Scatterplot showing the relationship between the change (Δ = T1–T0) in Total FIM scores and the change in Albumin in all patients. The linear regression line is shown as a dash-dotted line. Spearman’s correlation coefficient and *p*-value are also reported. Abbreviations: FIM, Functional Independence Measure.

**Figure 4:**
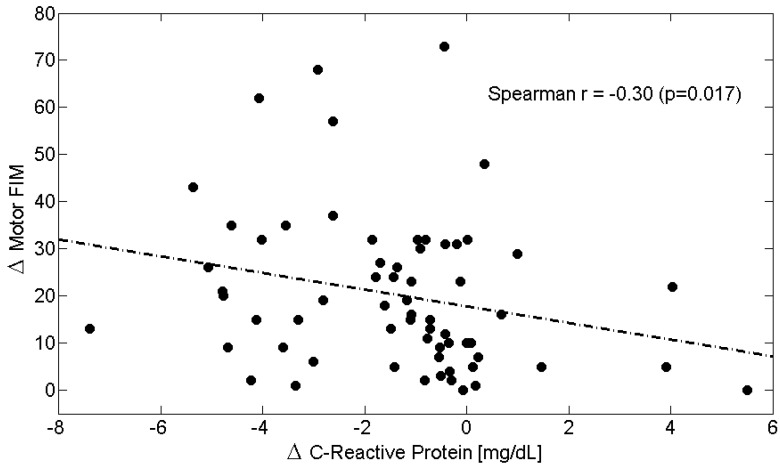
Scatterplot showing the relationship between the change (Δ = T1–T0) in Motor FIM scores and the change in C-Reactive Protein in all patients. The linear regression line is shown as a dash-dotted line. Spearman’s correlation coefficient and *p*-value are also reported. Abbreviations: FIM, Functional Independence Measure.

**Figure 5:**
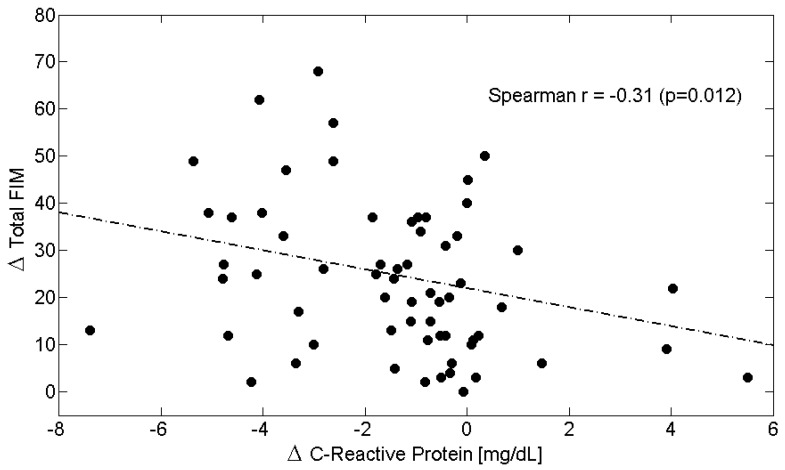
Scatterplot showing the relationship between the change (Δ = T1–T0) in Total FIM scores and the change in C-Reactive Protein in all patients. The linear regression line is shown as a dash-dotted line. Spearman’s correlation coefficient and *p*-value are also reported. Abbreviations: FIM, Functional Independence Measure.

Across the entire study population, longitudinal changes (Table 6) in circulating Alb levels were positively correlated with improvements in M-FIM (Figure 2) and T-FIM scores (Figure 3), while reductions in CRP levels were associated with improvements in M-FIM (Figure 4) and T-FIM scores (Figure 5).

**Figure 6:**
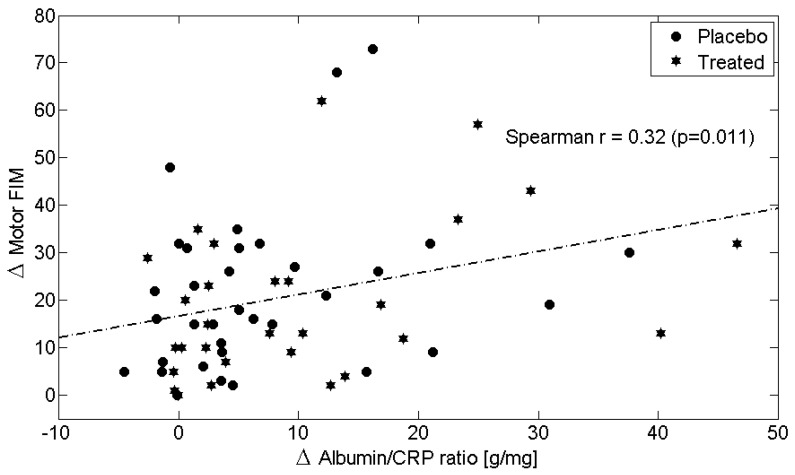
Scatterplot showing the relationship between the change (Δ = T1–T0) in Motor FIM scores and the change in the Albumin/C-Reactive Protein (CRP) ratio in both Placebo (circles) and Treated (stars) patients. The linear regression line for all patients is shown as a dash-dotted line. Spearman’s correlation coefficient and *p*-value (calculated for all patients) are also reported. Abbreviations: FIM, Functional Independence Measure; CRP, C-Reactive Protein.

Moreover, the Alb/CRP ratio showed a positive correlation with the recovery of neuromotor function (M-FIM) in both the EAA and placebo groups (Figure 6).

At discharge (Table 7), patients in both the placebo and EAA subgroups remained inflamed and hypoalbuminemic. The only variable showing a statistically significant difference between the groups was a reduction in haemoglobin, which was observed exclusively in the placebo arm (*p* = 0.041).

In summary, EAA supplementation significantly reduced systemic inflammation and improved Alb and prealbumin levels, although it did not fully normalize them.

## 4. Discussion

The study demonstrates that supplementation with a physiological dose of EAAs can attenuate systemic inflammation and increase circulating Alb levels in post-stroke patients. However, this dose was quantitatively and/or qualitatively inadequate to fully normalize these parameters. As a result, both placebo and EAA-treated patients were discharged from rehabilitation three months after the acute event still exhibiting residual inflammation and hypoalbuminemia. Nonetheless, the partial improvements observed with EAA supplementation should not be overlooked, especially considering the well-documented challenges of correcting inflammation and hypoalbuminemia through nutritional interventions in both acute [74] and subacute stroke patients [75]. In contrast, oral supplementation with BCAAs over 28 days has been shown to significantly improve albumin levels in patients with heart failure [76].

### 4.1. Baseline Inflammation and Low Alb Levels: Mechanisms and Potential Negative Influence on Functional Recovery

#### 4.1.1. Inflammation

This study supports previous findings that, following acute stroke, high circulating levels of CRP often persist [49,50]. This persistent elevation is likely driven by sustained increases in circulating interleukin-1 (IL-1) [47,48,51] and interleukin-6 (IL-6), a potent stimulator of CRP production [77]. A probable contributor to this chronic inflammatory state is the release of immune-active damage-associated molecular patterns (DAMPs) from the ischemic brain tissue [16,40], which perpetuate innate immune activation. Moreover, elevated CRP may itself exacerbate systemic inflammation through its pro-inflammatory properties [78]. CRP can promote insulin resistance [78], a known pro-inflammatory state [79] and contribute to endothelial cell inflammation [80]. Further research into the underlying mechanisms by which EAAs modulate inflammation and promote albumin synthesis may offer valuable insights. Reduced prealbumin levels are another factor likely contributing to an amplified inflammatory response. Physiological concentrations of prealbumin have been shown to inhibit IL-1 production by monocytes and endothelial cells [81], suggesting that hypo-pre albuminemia may remove a critical brake on inflammation. A major concern in post-stroke patients is that inflammation can induce metabolic changes in both the brain and skeletal muscle, potentially compromising the effectiveness of rehabilitation. In the brain, CRP has been shown to impair mRNA expression and stability, reduce cell survival, and inhibit apoptosis by decreasing NO production [82]. This reduction in NO is particularly detrimental, as endothelial NO is essential for maintaining vascular integrity. Its deficiency leads to endothelial dysfunction and impaired angiogenesis [82]. Beyond impairing cellular function, CRP directly damages cerebral circulation [83,84] by upregulating plasminogen activator inhibitor-1 (PAI-1) [85] and stimulating macrophages to release tissue factor, a potent procoagulant [86]. These changes contribute to a prothrombotic and pro-inflammatory environment.

The brain can also sustain damage due to nitrogen loss and decreased circulating AAs, often resulting from chronic inflammation [87,88,89,90,91,92]. Such AA dysmetabolism impairs both brain function and repair mechanisms due to the high demand for protein synthesis in brain tissue [93,94]. Importantly, the brain depends on circulating AAs, primarily released by skeletal muscle [95]. This dependency helps explain the observed association between sarcopenia and both brain atrophy and cognitive decline [96]. Inflammation further compromises rehabilitation outcomes by negatively impacting skeletal muscle. Specifically, the pro-inflammatory cytokine IL-6 induces rapid muscle wasting [97], compounded by hypercatabolism linked to reduced EAAs [13,92]. Notably, in subacute stroke patients, even the unaffected arm can undergo significant muscle catabolism—a condition shown to shift toward anabolism when an EAA supplement, identical to that used in the current study, is administered [13]. These inflammation-driven metabolic changes may contribute to several complications in stroke survivors, including brain atrophy [39], cognitive impairment [33], depression [18], and fatigue [17]. Each of these factors can hinder neurological recovery, prolong rehabilitation time, worsen patient outcomes, and increase the risk of post-stroke dementia [34].

#### 4.1.2. Low Circulating Alb

**Figure 7:**
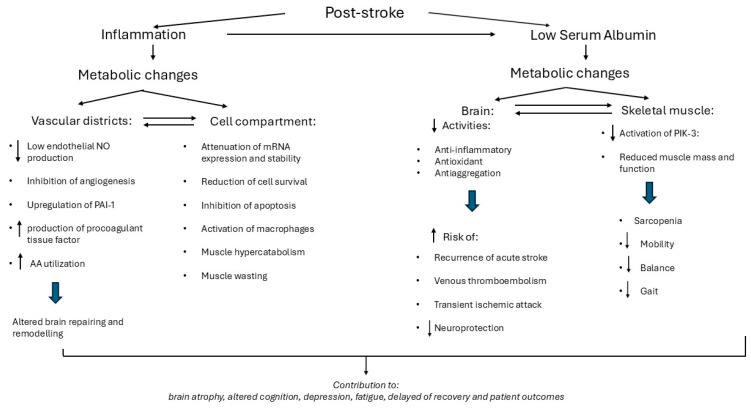
Schematic presentation of some metabolic effects primed by post-stroke inflammation on brain and skeletal muscle, contributing to persistent disability. Abbreviations: NO, nitric oxide; PAI-1, Plasminogen Activator Inhibitor Type 1; PIK-3, phosphoinositol-3-kinase.

During inflammation, low levels of Alb and prealbumin serve as negative acute-phase reactants, reflecting the body’s acute-phase response [98,99]. The inflammatory process—marked by elevated cytokines such as TNF-α, IL-1, and IL-6—shifts hepatic protein synthesis toward increased production of CRP at the expense of Alb. Additionally, inflammation-induced vascular permeability can lead to Alb extravasation, contributing further to hypoalbuminemia, which may persist until inflammation resolves [98]. Hypoalbuminemia, in turn, initiates a series of metabolic alterations that can delay patient rehabilitation and recovery. In the central nervous system, low Alb is linked to diminished anti-inflammatory [100], antioxidant [101,102,103], and anti-platelet aggregation activities [104]. These deficits are associated with increased risks of acute ischemic stroke recurrence [105], venous thromboembolism [106,107], and transient ischemic attack [108]. Consequently, low Alb levels reduce neuroprotection and are associated with increased long-term mortality [109]. Beyond neurological impacts, hypoalbuminemia is also strongly correlated with poor outcomes across both medical and surgical conditions [110,111,112,113,114]. Functionally, it may impair mobility, balance, and gait, as Alb supports muscle mass and strength through activation of phosphoinositol-3-kinase (PI3K) signalling pathways involved in muscle hypertrophy [115]. Therefore, the combined effects of systemic inflammation and hypoalbuminemia exert a detrimental influence on patients’ neurocognitive recovery. Figure 7 illustrates some of the inflammation-induced metabolic alterations in the brain and skeletal muscle that may negatively affect neurocognitive rehabilitation.

### 4.2. EAA-Induced Attenuation of Inflammation and Improvement of Hypoalbuminemia

#### 4.2.1. Attenuated Inflammation

**Figure 8:**
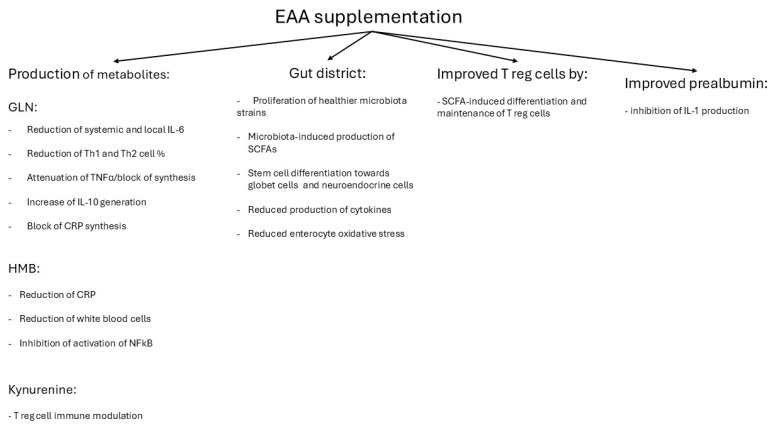
Plausible EAA-mechanisms potentially explaining the attenuation of systemic inflammation in the study patients. Abbreviations: EAA, essential amino acid; GLN, Glutamine; HMB, Hydroxymethylbutyrate; NF-κB, Nuclear Factor-Kappa B; SCFAs, Short-Chain Fatty Acids; T reg, T regulatory; IL-1, Interleukin-1.

During the rehabilitation phase, a general trend toward reduced systemic inflammation, improved immune response, as evidenced by a decreased N/L ratio, and correction of hypoalbuminemia was observed. This trend was significantly enhanced by supplementation with EAAs. EAAs exhibit anti-inflammatory effects through multiple mechanisms, including the activity of specific metabolites, suppression of pro-inflammatory cytokine production, modulation of gut microbiota, attenuation of intestinal inflammation, and enhancement of regulatory T cell (Treg) function (Figure 8).

Among the key metabolites generated during the catabolism of the essential amino acid leucine, glutamine (Gln) plays a prominent anti-inflammatory and immunomodulatory role [116,117]. Gln inhibits the synthesis of CRP, a strong promoter of myeloid-derived suppressor cell (MDSCs) formation [118,119]. Reductions in CRP levels similar to those observed in the current study have been reported following EAA administration in rehabilitative settings, such as dysphagic stroke patients [63] and elderly patients undergoing post-surgical rehabilitation after hip fracture [120]. Moreover, the observed increases in prealbumin levels following EAA supplementation may have further contributed to the anti-inflammatory effect, as prealbumin has been shown to inhibit IL-1 production by monocytes and endothelial cells [81]. Gln plays a crucial role in modulating inflammation, particularly under conditions of cytokine imbalance. It influences the production of pro-inflammatory cytokines such as IL-1β and TNF-α, thereby modulating the stress response [121,122]. In elderly sarcopenic patients with type 2 diabetes, Gln administration has been shown to reduce circulating TNF-α levels [62]. Furthermore, Gln supplementation decreases both systemic and localized IL-6 levels and reduces the proportions of Th1 and Th2 cells, thereby dampening excessive immune activation [123]. It also enhances anti-inflammatory responses by stimulating IL-10 production and attenuating TNF-α release from macrophages [123]. At the intestinal level, Gln reduces the release of pro-inflammatory cytokines from the mucosa [121] and promotes the differentiation of intestinal stem cells into goblet and neuroendocrine cells, contributing to improved mucosal integrity [124]. These combined actions result in decreased inflammation and oxidative stress [90].

Hydroxymethylbutyrate (HMB), another leucine-derived metabolite, has also been shown to exert anti-inflammatory effects. It reduces CRP and white blood cell counts [125] and suppresses excessive inflammatory responses by inhibiting nuclear factor-kappa B (NF-κB) activation [126]. BCAAs, including leucine, further contribute to anti-inflammatory activity by reshaping the gut microbiota. They promote the growth of beneficial microbial strains while suppressing pathogenic species. This microbial shift enhances the production of short-chain fatty acids (SCFAs), which support intestinal homeostasis. Specifically, butyrate promotes the differentiation of regulatory T (Treg) cells, while propionate maintains their function [127]. In clinical settings, EAA supplementation has been shown to eliminate gut inflammation, as demonstrated by the normalization of faecal calprotectin levels in patients with moderate-to-severe kidney disease [64]. Additionally, tryptophan (Trp), another EAA, contributes to Treg cell proliferation under inflammatory conditions through its catabolism to kynurenine, a pathway that enhances Treg-mediated immune regulation [90].

#### 4.2.2. Improvement of Hypoalbuminemia

**Figure 9:**
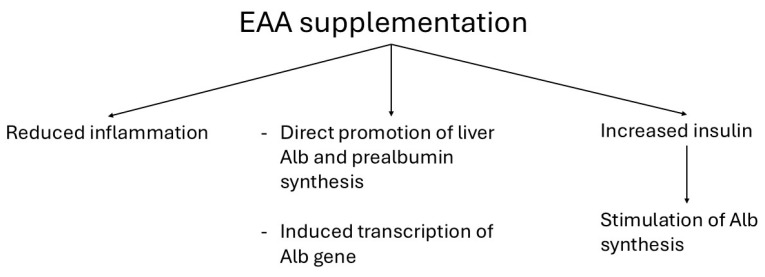
Some mechanisms underlying EAA-induced improvements of Alb and prealbumin of the study stroke survivors. Abbreviations: EAA, essential amino acid; Alb, Albumin.

EAA supplementation may improve hypoalbuminemia both indirectly, by reducing inflammation, and directly, by promoting Alb synthesis. Directly, EAAs stimulate hepatic Alb production through enhanced protein synthesis [128,129] and by inducing transcription of the Alb gene [130]. Indirectly, EAAs may also enhance Alb synthesis by increasing circulating insulin levels, which are known to stimulate hepatic Alb production [131] (Figure 9). Furthermore, the presence of Trp in the EAA formulation likely contributed to improved Alb synthesis, as Trp is an essential precursor for Alb production [132].

### 4.3. After 2-Month Rehabilitation: Relationship Between Metabolic Variables and Functional Recovery

At three months post-acute stroke and following two months of rehabilitation, patients, considered as a whole group, were discharged with persistent systemic inflammation and hypoalbuminemia, despite achieving normal glucose control. Importantly, they continued to experience significant overall disability, as reflected by T-FIM scores. While correlation does not imply causation, we believe the observed associations between Alb and both M-FIM and T-FIM scores (positive correlation), as well as between CRP and M-FIM, T-FIM scores (negative correlation), are unlikely to be coincidental. Prior research has demonstrated the critical influence of both Alb and CRP on the injured brain (as discussed in the Introduction). In the present study, the positive correlation between the Alb/CRP ratio and improvement in neuromotor function suggests that increasing Alb levels may play a more pivotal role in neurological recovery than merely reducing inflammation. Notably, Alb has been shown to be a stronger prognostic indicator of survival than inflammatory markers in emergency department settings [133]. Additionally, a recent study reported an association between early cardiovascular events and mortality in patients with acute ischemic stroke [134], while another investigation linked long-term mortality to both low and low-normal Alb concentrations [109].

### 4.4. Potential Factors Limiting a Full Response to EAA Supplementation

The incomplete response of both systemic inflammation and hypoalbuminemia to EAA supplementation may be attributed to potential inadequacies in the composition and/or dosage of the EAA formula, relative to the complex demands of the brain and peripheral tissues. The ischemic brain exhibits a heightened requirement for EAAs to support repair and remodelling processes [7,135,136], as well as for the synthesis of neurotransmitters [92,137]. Beyond the brain, extraneural tissues, particularly the gut and skeletal muscle, play critical roles in amino acid metabolism following stroke. Stroke-induced intestinal dysbiosis [138] may impair amino acid absorption across the gut wall and enhance amino acid deamination, ultimately reducing hepatic availability of amino acids and thus limiting Alb synthesis [139]. Simultaneously, skeletal muscle tissue, a primary consumer of BCAAs, likely sequestered a significant portion of the supplemented EAAs, as BCAAs serve as a vital energy substrate for muscle [117]. In fact, a previous study in rehabilitative stroke patients demonstrated that two months of EAA supplementation shifted muscle metabolism from a hypercatabolic to an anabolic state [13]. Another critical limiting factor for Alb synthesis may be the low Trp content in the EAA formulation, only 50 mg per packet, which represents approximately 7.7% of the amount typically provided by a standard diet (data not published). Trp is the rate-limiting amino acid for Alb synthesis [132,140]. Notably, earlier research documented reduced plasma Trp levels in rehabilitative stroke patients [92], suggesting that the insufficient Trp content in the formula may have been especially detrimental in this patient population.

### 4.5. Limitations

This study has several limitations. First, serum levels of mercaptoalbumin (the reduced form of Alb) and non-mercaptoalbumin (the oxidized form) were not measured due to technical constraints. The ratio of reduced to oxidized Alb is metabolically significant, particularly in the context of tissue repair. Oxidized Alb exhibits diminished drug-binding capacity and impaired radical scavenging activity, thereby reducing its protective functions [130,141]. Additionally, oxidized Alb can promote inflammation by activating neutrophils [142] and mononuclear cells [143], potentially hindering tissue healing. Alb oxidation also increases the molecule’s susceptibility to proteolytic degradation, leading to accelerated Alb breakdown [130]. Another factor that may limit albumin synthesis is intestinal bacterial overgrowth, which can impair amino acid absorption and increase deamination within the gut, thereby reducing amino acid availability for hepatic Alb production [139]. Given these considerations, future studies investigating the role of circulating Alb in tissue repair should account for at least these factors to better understand the therapeutic potential and metabolic impact of Alb in post-stroke recovery.

### 4.6. Future Studies

Future research should aim to determine the optimal tryptophan content in EAA formulations and/or the most effective dosing regimen to maximize the resolution of both inflammation and hypoalbuminemia—two key factors that may significantly enhance neurofunctional recovery in post-stroke patients. It would be of interest to determine whether the administration of protein hydrolysates in stroke patients could yield similar outcomes to those observed with essential amino acid (EAA) supplementation.

Future studies with larger sample sizes are needed to confirm the current findings and improve their generalizability.

## 5. Conclusions

This study demonstrates the feasibility of improving post-stroke inflammation and hypoalbuminemia through supplementation with a physiological dose of EAAs. However, the findings also highlight the limitations of the current EAA formulation, which proved insufficient to fully resolve systemic inflammation and correct hypoalbuminemia.

## Figures and Tables

**Table 1:** Nutrition composition of an individual packet of supplements, containing 4.2 g of essential amino acids, used in this study *.

Mixture Substrates:	5.1 g
L-leucine ^§^	1200 mg
L-lysine ^§^	900 mg
L-threonine ^§^	700 mg
L-isoleucine ^§^	600 mg
L-valine ^§^	600 mg
L-cystine	150 mg
L-histidine	150 mg
L-phenylalanine ^§^	100 mg
L-methionine ^§^	50 mg
L-tryptophan ^§^	50 mg
Vitamin B6	0.85 mg
Vitamin B1	0.70 mg
Citric acid	409 mg
Succinic acid	102.5 mg
Malic acid	102.5 mg

* Treated patients were given 2 packets daily (8.4 g essential amino acids). ^§^ Indicates essential amino acids.

**Table 2:** Demographic, anthropometric characteristics and comorbidities of the patients after randomization (Placebo and EAA groups).

Variable	Placebo (N = 34)	EAA (N = 30)	*p* Value
**Demographic**			
Age (yrs) *	71.1 ± 13.7	76.4 ± 9.9	0.09
Sex (Males,%) ^§^	18 (52.9%)	12 (40.0%)	0.30
**Anthropometric**			
Body weight (kg) *	65.8 ± 12.0	66.5 ± 17.4	0.84
Body Mass Index (BMI, kg/m^2^) *	24.0 ± 3.8	24.1 ± 4.9	0.97
**Comorbidities**			
Hypertension ^§^	27 (79.4%)	23 (76.7%)	0.79
Atrial fibrillation ^§^	11 (32.4%)	9 (30.0%)	0.84
Ischemic heart disease ^§^	4 (11.8%)	5 (16.7%)	0.57
Dyslipidemia ^§^	12 (35.3%)	10 (33.3%)	0.87
Diabetes mellitus ^§^	8 (23.5%)	9 (30.0%)	0.56
**Drugs taken upon admission or during hospitalization**			
ACE inhibitors ^§^	20 (58.8%)	17 (56.7%)	0.86
Calcium antagonists ^§^	10 (29.4%)	13 (43.3%)	0.25
Beta-blockers ^§^	14 (41.2%)	11 (36.7%)	0.71
Antihypertensives ^§^	27 (79.4%)	23 (76.7%)	0.79
Antiaggregants or anticoagulants ^§^	22 (64.7%)	21 (70.0%)	0.65
Lipid-lowering agents ^§^	14 (41.2%)	12 (40.0%)	0.92
Hypoglycemic agents ^§^	6 (17.6%)	8 (26.7%)	0.38
Antidepressants ^§^	15 (44.1%)	17 (56.7%)	0.32
Neuroleptics (quetiapine) ^§^	8 (23.5%)	9 (30.0%)	0.56
Antidepressant + quetiapine ^§^	12 (35.3%)	9 (30.0%)	0.65
Antibiotics (at admission) ^§^	20 (58.8%)	11 (36.7%)	0.08

*: *p* values are from unpaired *t*-test; ^§^: *p* values are from chi-square test. Abbreviations: EAA, Essential Amino Acid; ACE, angiotensin converting enzyme.

**Table 3:** Clinical characteristics and bio humoral variables in the stroke population at admission to the rehabilitation ward.

Variable	Stroke Population T0 (N = 64)
**Clinical**	
*Aetiology (% patients)*	
Ischemic	82.8
Haemorrhagic	17.2
*Ischemic stroke location (% patients)*	
LACI	15.1
PACI	30.2
POCI	20.8
TACI	34.0
*Route of feeding (% patients)*	
Oral	79.7
PEG	20.3
**Biohumoral variables**	
Erythrosedimentation rate (NV: <15 mm/1st h)	49.40 ± 27.21
Haemoglobin (NV: M 13.2–17.3 g/dL; F 11.7–15.5 g/dL)	12.31 ± 1.82
Blood urea (NV: 16–40 mg/dL)	47.63 ± 25.92
Creatinine (NV: M 0.73–1.18 mg/dL; F 0.55–1.02 mg/dL)	1.00 ± 0.39
Glucose (NV: 70–115 mg/dL)	104.4 ± 28.1
Total white blood cells (TWBC, NV: 4000–10,000/µL)	7949 ± 2065
Neutrophils % TWBC	64.85 ± 10.81
Neutrophil count (NV: 2000–8000/µL)	6102 ± 1727
Lymphocytes % TWBC	22.59 ± 9.45
Lymphocyte count (NV: 1500–4000/µL)	1677 ± 468
Monocytes % TWBC	8.93 ± 2.08
Monocyte count (NV: 100–1000/µL)	735.1 ± 238.5
Neutrophil/Lymphocyte ratio (NV: 1.5–3)	3.50 ± 1.67
Albumin (NV: 3.5–4.76 g/dL)	3.08 ± 0.51
Prealbumin (NV: 18–32 mg/dL)	17.67 ± 5.33
Fibrinogen (NV: 230–550 mg/dL)	455.7 ± 114.8
Transferrin (NV: 202–364 mg/dL)	200.4 ± 38.8
C-Reactive Protein (CRP: NV < 0.5 mg/dL)	2.48 ± 1.99
Albumin/CRP ratio (g/mg)	2.35 ± 1.72
**Total-FIM** (NV: 18–126 points)	38.73 ± 19.90
**Motor-FIM** (NV: 13–91 points)	21.97 ± 12.36
**Cognitive-FIM** (NV: 5–35 points)	16.73 ± 10.65

Abbreviations: T0, Time 0; NV, normal values; F, female; M, male; LACI, lacunar infarction; PACI, partial anterior circulation infarction; POCI, posterior circulation infarction; TACI, total anterior circulation infarction; PEG, percutaneous endoscopy gastrostomy; TWBC, Total White Blood Cells; FIM, Functional Independence Measure.

**Table 4:** Comparisons in baseline (T0) values of bio humoral variables and Functional Independence Measure in Placebo and EAA groups.

Variable	Placebo (N = 34)	EAA (N = 30)	*p*-Value
**Bio humoral**			
C-Reactive Protein (CRP: NV < 0.5 mg/dL)	2.13 ± 1.82	2.89 ± 2.12	0.13
Albumin (NV: 3.5–4.76 g/dL)	3.10 ± 0.46	3.07 ± 0.57	0.82
Prealbumin (NV: 18–32 mg/dL)	18.3 ± 6.2	16.9 ± 3.9	0.28
Albumin/CRP ratio (g/mg)	2.58 ± 1.68	2.03 ± 1.72	0.20
Body Mass Index (BMI, kg/m^2^)	24.0 ± 3.8	24.1 ± 4.9	0.97
Body weight (kg)	65.8 ± 12.0	66.5 ± 17.4	0.84
Haemoglobin (NV: M 13.2–17.3 g/dL; F 11.7–15.5 g/dL)	12.3 ± 1.6	12.4 ± 2.0	0.83
Blood urea (NV: 16–40 mg/dL)	51.2 ± 23.9	43.2 ± 27.4	0.22
Creatinine (NV: M 0.73–1.18 mg/dL; F 0.55–1.02 mg/dL)	1.06 ± 0.46	0.93 ± 0.29	0.19
Glucose (NV: 70–115 mg/dL)	101.5 ± 17.3	115.1 ± 44.1	0.10
Erythrosedimentation rate (NV: <15 mm/1st h)	49.5 ± 25.2	52.3 ± 32.2	0.72
Total white blood cells (TWBC, NV: 4000–10,000/µL)	7900 ± 2306	8004 ± 1792	0.84
Neutrophils % TWBC	66.0 ± 9.9	63.5 ± 11.7	0.35
Neutrophil count (NV: 2000–8000/µL)	6529 ± 1771	5675 ± 1643	0.23
Lymphocytes % TWBC	21.6 ± 9.3	23.8 ± 9.6	0.36
Lymphocyte count (NV: 1500–4000/µL)	1652 ± 501	1701 ± 453	0.80
Monocytes % TWBC	8.48 ± 2.04	9.44 ± 2.04	0.065
Monocyte count (NV: 100–1000/µL)	836 ± 295	657 ± 161	0.14
Fibrinogen (NV: 230–550 mg/dL)	445 ± 111	476 ± 124	0.31
**Total-FIM** (NV: 18–126 points)	42.3 ± 20.5	34.7 ± 18.7	0.13
**Motor-FIM** (NV: 13–91 points)	24.0 ± 13.9	19.7 ± 10.2	0.17
**Cognitive-FIM** (NV: 5–35 points)	18.2 ± 10.3	15.0 ± 11.0	0.23

*p* values are from unpaired *t*-test. Abbreviations: EAA, Essential Amino Acid; TWBC, Total White Blood Cells; CRP, C-Reactive Protein; F, female; M, male; FIM, Functional Independence Measure; T0, Time 0; NV, normal values.

**Table 5:** Time courses of bio humoral variables in Placebo and EAA groups.

Variable	Δ Placebo (N = 34)	Δ EAA (N = 30)	*p*-Value
**Bio humoral**			
Albumin (NV: 3.5–4.76 g/dL)	0.17 ± 0.44	0.36 ± 0.38	0.033
C-Reactive Protein (CRP: NV < 0.5 mg/dL)	−0.86 ± 2.25	−2.04 ± 2.15	0.036
Albumin/CRP ratio (g/mg)	7.21 ± 9.59	10.28 ± 12.44	0.27
Body Mass Index (BMI, kg/m^2^)	−0.36 ± 1.15	−0.27 ± 1.14	0.75
Body weight (kg)	−1.07 ± 3.33	−0.87 ± 3.14	0.81
Haemoglobin (NV: M 13.2–17.3 g/dL; F 11.7–15.5 g/dL)	−0.63 ± 1.66	0.05 ± 1.32	0.08
Blood urea (NV: 16–40 mg/dL)	−7.9 ± 20.1	−2.8 ± 28.5	0.41
Creatinine (NV: M 0.73–1.18 mg/dL; F 0.55–1.02 mg/dL)	−0.00 ± 0.26	−0.01 ± 0.19	0.92
Glucose (NV: 70–115 mg/dL)	−6.3 ± 12.8	−15.5 ± 28.2	0.12
Erythrosedimentation rate (NV: <15 mm/1st h)	−8.5 ± 25.2	−14.2 ± 20.2	0.44
Total white blood cells (TWBC, NV: 4000–10,000/µL)	−1676 ± 1852	−1256 ± 2562	0.45
Neutrophils % TWBC	−7.60 ± 7.84	−6.62 ± 10.86	0.68
Neutrophil count (NV: 2000–8000/µL)	−2061 ± 1864	−1085 ± 2864	0.33
Lymphocytes % TWBC	7.13 ± 7.63	7.08 ± 9.43	0.98
Lymphocyte count (NV: 1500–4000/µL)	305 ± 314	177 ± 189	0.24
Monocytes % TWBC	−0.04 ± 2.09	−0.11 ± 2.36	0.90
Monocyte count (NV: 100–1000/µL)	−159 ± 321	33 ± 369	0.29
Prealbumin (NV: 18–32 mg/dL)	0.61 ± 4.19	3.08 ± 5.65	0.05
Fibrinogen (NV: 230–550 mg/dL)	−58.8 ± 93.8	−62.8 ± 122.3	0.90
**Total-FIM** (NV: 18–126 points)	26.4 ± 19.1	23.0 ± 17.3	0.47
**Motor-FIM** (NV: 13–91 points)	21.6 ± 16.9	18.8 ± 16.2	0.51
**Cognitive-FIM** (NV: 5–35 points)	4.88 ± 5.93	4.23 ± 6.34	0.67

*p*-values are from unpaired *t*-test for the comparison between the two groups (placebo vs. EAA) of the deltas (difference between the values at T1 and the values at T0). Abbreviations: EAA, Essential Amino Acid; TWBC, Total White Blood Cells; CRP, C-Reactive Protein; NV, normal values; F, female; M, male; T0, Time 0; T1, Time 1; FIM, Functional Independence Measure.

**Table 6:** Correlations (Spearman’s r) between biochemical variables and motor-cognitive functions.

Δ_s_	C-Reactive Protein (CRP)	Albumin	Albumin/CRP Ratio	Total-FIM	Motor-FIM	Cognitive-FIM
**C-Reactive Protein (CRP)**	1.00	−0.32 ^†^	−0.55 ^‡^	−0.31 ^^^	−0.30 ^^^	−0.11
**Albumin**	−0.32 ^†^	1.00	0.32 ^^^	0.32 ^^^	0.32 ^^^	0.03
**Albumin/CRP ratio**	−0.55 ^‡^	0.32 ^^^	1.00	0.28 ^^^	0.32 ^^^	−0.01
**Total-FIM**	−0.31 ^^^	0.32 ^^^	0.28 ^^^	1.00	0.93 ^‡^	0.39 ^†^
**Motor-FIM**	−0.30 ^^^	0.32 ^^^	0.32 ^^^	0.93 ^‡^	1.00	0.13
**Cognitive-FIM**	−0.11	0.03	−0.01	0.39 ^†^	0.13	1.00

^: *p* < 0.05; ^†^: *p* < 0.01; ^‡^: *p* < 0.001. Abbreviations: Δ_s_, overtime changes; CRP, C-Reactive Protein; FIM, Functional Independence Measure.

**Table 7:** Comparisons at discharge (T1) values of bio humoral variables and Functional Independence Measure in Placebo and EAA groups.

Variable	Placebo (N = 34)	EAA (N = 30)	*p*-Value
**Bio humoral**			
Albumin (NV: 3.5–4.76 g/dL)	3.26 ± 0.42	3.42 ± 0.47	0.18
C-Reactive Protein (CRP: NV < 0.5 mg/dL)	1.26 ± 2.40	0.85 ± 1.05	0.38
Albumin/CRP ratio (g/mg)	9.8 ± 9.8	12.3 ± 12.4	0.36
Body Mass Index (BMI, kg/m^2^)	23.6 ± 4.0	23.8 ± 4.6	0.90
Body weight (kg)	64.7 ± 12.0	65.6 ± 16.2	0.79
Haemoglobin (NV: M 13.2–17.3 g/dL; F 11.7–15.5 g/dL)	11.6 ± 1.1	12.4 ± 1.8	0.041
Blood urea (NV: 16–40 mg/dL)	43.3 ± 20.9	40.6 ± 19.8	0.60
Creatinine (NV: M 0.73–1.18 mg/dL; F 0.55–1.02 mg/dL)	1.06 ± 0.38	0.92 ± 0.25	0.10
Glucose (NV: 70–115 mg/dL)	92.7 ± 13.5	95.4 ± 20.8	0.55
Erythrosedimentation rate (NV: <15 mm/1st h)	40.5 ± 23.9	33.0 ± 23.9	0.29
Total white blood cells (TWBC, NV: 4000–10,000/µL)	6223 ± 2062	6748 ± 2461	0.36
Neutrophils % TWBC	58.4 ± 9.0	56.9 ± 10.4	0.52
Neutrophil count (NV: 2000–8000/µL)	4469 ± 2249	4590 ± 2628	0.90
Lymphocytes % TWBC	28.7 ± 9.2	30.8 ± 9.9	0.37
Lymphocyte count (NV: 1500–4000/µL)	1957 ± 630	1878 ± 602	0.76
Monocytes % TWBC	8.44 ± 2.09	9.32 ± 1.86	0.08
Monocyte count (NV: 100–1000/µL)	677 ± 374	690 ± 507	0.96
Prealbumin (NV: 18–32 mg/dL)	19.1 ± 5.4	19.8 ± 5.6	0.58
Fibrinogen (NV: 230–550 mg/dL)	380 ± 59	408 ± 103	0.23
**Total-FIM** (NV: 18–126 points)	68.6 ± 27.7	57.8 ± 28.6	0.13
**Motor-FIM** (NV: 13–91 points)	45.5 ± 21.3	38.5 ± 20.0	0.18
**Cognitive-FIM** (NV: 5–35 points)	23.1 ± 9.4	19.3 ± 14.6	0.21

*p* values are from unpaired *t*-test. Abbreviations: EAA, Essential Amino Acid; TWBC, Total White Blood Cell Count; CRP, C-Reactive Protein; FIM, Functional Independence Measure; F, female; M, male; T0, Time 0; NV, normal values.

## Data Availability

The data that support the findings of this study are available from the corresponding author upon reasonable request.

## Abbreviations

The following abbreviations are used in this manuscript:

Alb	Albumin
FIM	Functional Independence Measure
EAAs	Essential Amino Acids
BCAAs	Branched Chain Amino Acids
BMI	Body Mass Index
BW	Body Weight
N.V.	Normal Value
T0	Time 0
T1	Time 1
CRP	C-Reactive Protein
TWBC	Total White Blood Cell Count
T-FIM	Total Functional Independence Measure
M-FIM	Motor Functional Independence Measure
C-FIM	Cognitive Functional Independence Measure
Plac	Placebo group
N/L ratio	Neutrophil/lymphocyte ratio
PSI	Post stroke inflammation
